# Mapping Intellectual Structures and Research Hotspots of Triple Negative Breast Cancer: A Bibliometric Analysis

**DOI:** 10.3389/fonc.2021.689553

**Published:** 2022-01-03

**Authors:** Kai-jun Hao, Xiao Jia, Wen-ting Dai, Ze-min Huo, Hua-qiang Zhang, Jing-wei Liu, Xiao-bing Wang

**Affiliations:** ^1^ Department of Plastic Surgery, The First Affiliated Hospital of Shanxi Medical University, Taiyuan, China; ^2^ Key Laboratory of Evidence Science, China University of Political Science and Law, Ministry of Education, Beijing, China

**Keywords:** triple negative breast cancer, bibliometric analysis, co-word analysis, co-citation analysis, research hotspots

## Abstract

**Background:**

Triple negative breast cancer (TNBC) is a highly heterogeneous breast cancer subtype with a poor prognosis due to its extremely aggressive nature and lack of effective treatment options. This study aims to summarize the current hotspots of TNBC research and evaluate the TNBC research trends, both qualitatively and quantitatively.

**Methods:**

Scientific publications of TNBC-related studies from January 1, 2010 to October 17, 2020 were obtained from the Web of Science database. The BICOMB software was used to obtain the high-frequency keywords layout. The gCLUTO was used to produce a biclustering analysis on the binary matrix of word-paper. The co-occurrence and collaboration analysis between authors, countries, institutions, and keywords were performed by VOSviewer software. Keyword burst detection was performed by CiteSpace.

**Results:**

A total of 12,429 articles related to TNBC were identified. During 2010-2020, the most productive country/region and institution in TNBC field was the USA and The University of Texas MD Anderson Cancer Center, respectively. Cancer Research, Journal of Clinical Oncology, and Annals of Oncology were the first three periodicals with maximum publications in TNBC research. Eight research hotspots of TNBC were identified by co-word analysis. In the core hotspots, research on neoadjuvant chemotherapy, paclitaxel therapy, and molecular typing of TNBC is relatively mature. Research on immunotherapy and PARP inhibitor for TNBC is not yet mature but is the current focus of this field. Burst detection of keywords showed that studies on TNBC proteins and receptors, immunotherapy, target, and tumor cell migration showed bursts in recent three years.

**Conclusion:**

The current study revealed that TNBC studies are growing. Attention should be paid to the latest hotspots, such as immunotherapy, PARP inhibitors, target, and TNBC proteins and receptors.

## Introduction

Breast cancer is one of the most common malignancies in women worldwide, and its mortality rate ranks second in cancer-related deaths (1). Triple negative breast cancer (TNBC) is a subtype of breast cancer where there is reduced expression of estrogen receptor (ER), progesterone receptor (PR), and human epidermal growth factor receptor 2 (HER2) receptor. TNBC accounts for 20% of all newly-diagnosed breast cancers (2). Among highly heterogeneous diseases, TNBC has highly invasive biological characteristics and earlier age of onset, and early recurrence and distant metastasis are common (3, 4). Most TNBC treatments are limited as therapeutic targets have not been elucidated (5). Adjuvant chemotherapy is currently the standard treatment for TNBC, but the optimal chemotherapy regimen is still controversial due to drug resistance and tolerance issues (3, 6). Therefore, it is urgent to find specific therapeutic targets to improve the clinical outcomes, which has become a hotspot of TNBC research (7).

Bibliometrics is a quantitative analysis method that uses co-word and co-citation analyses of existing research to help scholars quickly identify popular themes and emerging trends in a particular field of study (8, 9). Among them, CiteSpace, VOSviewer, Bibliographic Items Co-occurrence Matrix Builder (BICOMB), and BibExcel are commonly used tools for bibliometric analysis and visualization (10). In recent years, many scholars have conducted bibliometric analysis on diseases, such as coronavirus disease, Alzheimer’s disease, pancreatic cancer, and obesity (11–15). However, there is no bibliometric study on TNBC. Therefore, we collated the last ten years’ scientific publications on TNBC from the Web of Science (WoS) database and systematically summarized the studies using Citespace, VOSviewer, BICOMB, and BibExcel software. We present the field structure and the development of knowledge and highlight the research hotspots and future directions in this field to provide a reference for further clinical research on TNBC.

## Methods

### Data Sources and Retrieval Strategies

The WoS core collection was used as the data source. The retrieval strategy was as follows: subject words = triple-negative breast cancer or subject words = triple negative breast cancer, literature type = article or review, language = English, year = 2010–2020. A total of 12,429 studies were retrieved. All records and references were downloaded in a TXT format. To avoid deviation caused by the frequent update of the database, all literature retrievals and data extractions were finalized on October 17, 2020 and introduced into the Bibliometrics analysis software for further analysis.

### Co-Citation Analysis

CiteSpace software was developed by Dr. Chaomei Chen using Java. It is mainly applied to visualization analysis of scientific literature, which is usually applicable to “co-citation analysis” of large volumes of literature data in a particular field of study. The settings were as follows: from 2010 to 2020, years per slice = 1, and the top 50 of the most cited papers in a year per individual network. Based on our research goals, each node represented a citation, with the larger size of the node denoting a greater frequency (16, 17). The author co-citation and literature co-citation networks were constructed, and keyword burst detection was performed. Additionally, the java program VOSviewer (Leiden University, Leiden, Netherlands) was used to visualize the cooperative networks and keyword co-occurrence between countries/regions and institutions (18).

### Co-Word Analysis

The TXT files were imported to BICOMB (14, 19) for fetching high-frequency keywords. Based on this, binary matrices of word-paper and co-word matrices of high-frequency words were generated. gCLUTO was used to produce a biclustering analysis on the binary matrix of word-paper to determine the research hotspots of TNBC (20, 21). To improve the display of the clustering results, visualized mountain maps and heat maps were generated as per the results of the biclustering analysis.

### Strategic Diagram Analysis

In 1998, John Law proposed a series of strategic diagrams to reveal the current development situation on each research topic in specific fields and predict their future development trends (22). Using Excel, we imported the cluster information from gCLUTO into the co-word matrix and calculated the intra-class and inter-class link averages for each hotspot category. The centrality and density were then calculated. Subsequently, a two-dimensional strategic diagram was established. The X-axis and Y-axis represent the centrality and the density, respectively. Among them, the centrality was a criterion of interaction among various clusters, and with greater centrality, the cluster had a greater central tendency in a research field. The density represents the strength of the internal connections of a cluster, which is used to measure the ability to maintain the internal integration within the cluster.

## Results

### Annual Analysis of Publications

**Figure 1:**
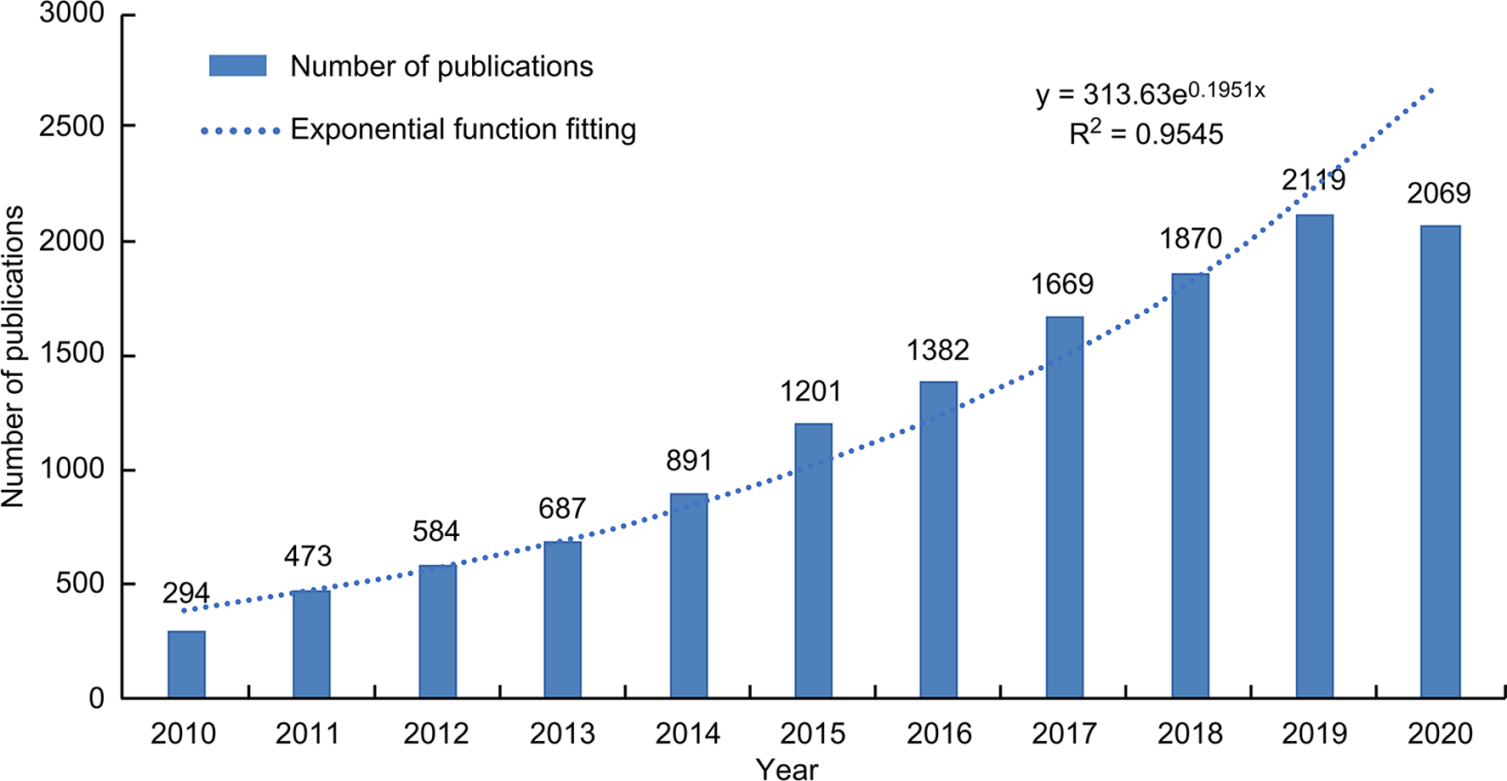
The trend of TNBC research from 2010 to 2020.

Between 2010 and October 17, 2020, 12,429 TNBC articles were published and listed on WoS (**Figure 1**). The cumulative number of posts related to TNBC has maintained a rapid growth every year since 2010. The annual growth trend is in line with the fitting curve y=313.63e0.1951X (R2 = 0.9545). This indicates that TNBC is arousing increasing attention and has clinical significance and development potential.

### Distribution Characteristics of Countries/Regions and Institutions

**Figure 2:**
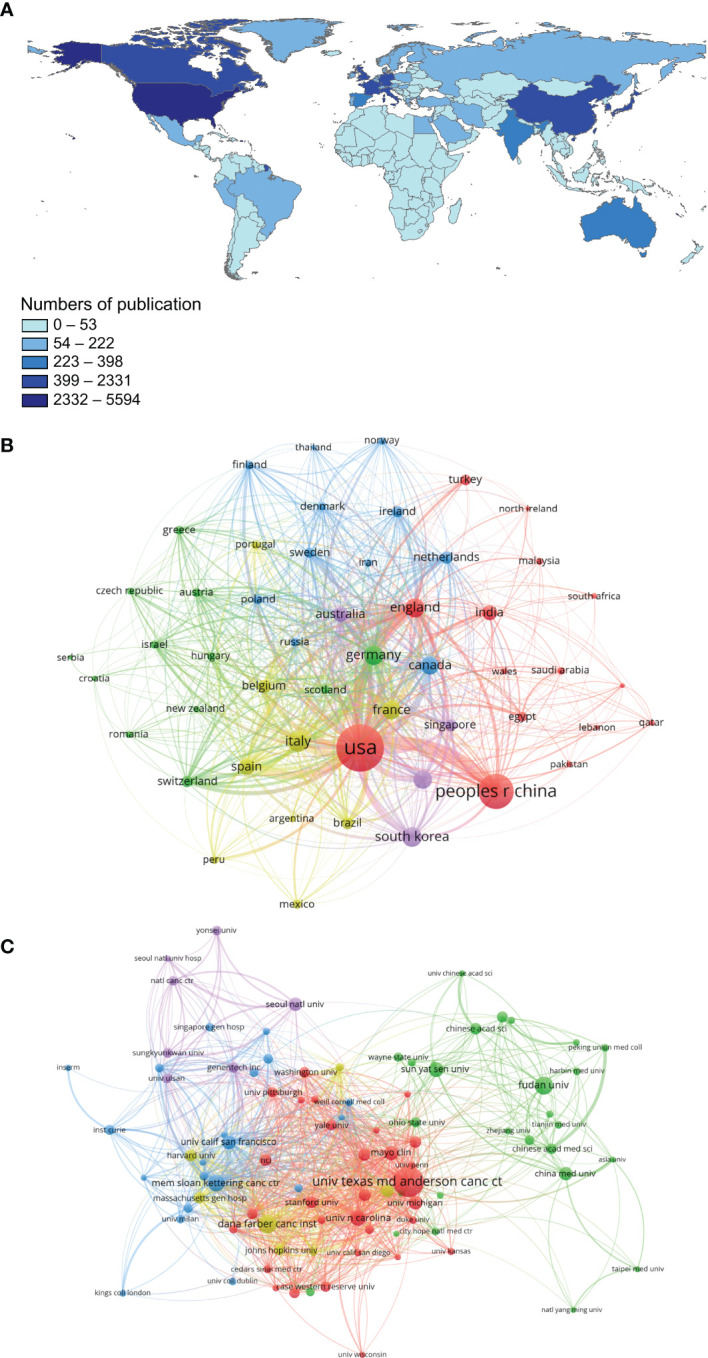
Main countries/regions and institutions of TNBC research and their interrelationships. **(A)** Countries/Regions distribution of TNBC-related research results; **(B)** A visualization network of collaboration between countries/regions in TNBC research; **(C)** A visualization network of collaboration among institutions in TNBC research.

Since 2010, 101 countries/regions have participated in TNBC studies. The maps created by CiteSpace and Google Earth have shown the distributions and numbers of countries/regions of publications (**Figure 2A**). The top 10 countries published a total of 12,327 articles over the past decade. The United States had the most publications (n=5420), followed by China (n=2555), Italy (n=643), and South Korea (n=634). Centrality was used to evaluate the importance of nodes in a network. **Table 1** revealed that the United States also had the highest centrality (0.27). This suggests that the United States is the most prolific and influential country in TNBC research. According to the collaborative visualization network of publishing countries/regions (**Figure 2B**), the United States and China are the two largest network nodes located at the central connection point of the collaborative relationship map, i.e., they are most closely connected with other major publishing countries. In the collaborative network map, the lines between China and the United States are the widest, indicating the large partnership community between the two countries for TNBC research. In contrast, the cooperative ties among other countries could be strengthened.

**Table 1:** The main countries, regions, and institutions contributing to publications in TNBC research.

Rank	Country/Region	Article Counts	Proportion	Centrality	Institutions	Article Counts	Proportion	H Index	Total Number of Citations	Average Number of Citations
1	USA	5420	44.0%	0.27	Univ Texas Md Anderson Canc Ctr	478	5.45%	52	10830	22.7
2	People’s Republic of China	2555	20.7%	0.01	Dana Farber Canc Inst	239	2.72%	53	9382	39.3
3	Italy	643	5.22%	0.1	Fudan Univ	230	2.62%	27	2449	10.7
4	South Korea	634	5.14%	0.01	Mem Sloan Kettering Canc Ctr	204	2.33%	33	4849	23.8
5	Germany	583	4.73%	0.18	Univ N Carolina	190	2.17%	39	8027	42.2
6	England	560	4.54%	0.11	Sun Yat Sen Univ	176	2.01%	30	2606	14.8
7	Japan	546	4.43%	0.06	Mayo Clin	168	1.92%	32	3863	23.0
8	France	522	4.23%	0.17	Univ Calif San Francisco	158	1.80%	40	6178	39.1
9	Canada	482	3.91%	0.12	Univ Michigan	157	1.79%	26	2348	15.0
10	Spain	382	3.10%	0.06	China Med Univ	153	1.74%	23	2086	13.6

A total of 9,187 institutions participated in the TNBC study. The University of Texas MD Anderson Cancer Center published the maximum number of papers (n=478), followed by Dana-Farber Cancer Institute (n=239), Fudan University (n=230), and Memorial Sloan Kettering Cancer Center (n=204). The H index is primarily used to evaluate the comprehensive influential power of a specific institution. The results from **Table 1** show that although Chinese institutions were high on the ranking list of total publications, their H indices and total and average numbers of citations were significantly lower. Thus, although China has been relatively active in TNBC research in recent years and produced numerous papers, its global attention and international influence are still low. Further, we used VOSviewer software to analyze the collaborative visualization networks among these institutions (**Figure 2C**). The results showed a scattered distribution and insufficient cooperation among the international institutions.

### Journal Analysis

Since 2010, 1,056 journals have published articles on TNBC. We identified the top 10 most popular journals with 4,359 published articles over the past decade, accounting for 35.01% of all articles (**Table 2**). Thus, emphasizing posts from these key journals helped us keep abreast of the latest trends. Cancer Research, Journal of Clinical Oncology, and Annals of Oncology were not only among the first three periodicals with maximum publications but also among the first three journals in the Impact Factor list. They were classified as Q1 by the Journal Citation Reports standard and were important sources of knowledge for TNBC. The analysis of the core author and intellectual basis of the research field is shown in **Supplementary Material** (10, 16, 17, 23, 24). (**Supplementary Figure 1** and **Supplementary Table 1**).

**Table 2:** The top 10 highly-productive journals in TNBC research.

Rank	Journal	Number of publications	Proportion	IF [2019]	Quartile in category [2019]
1	Cancer Research	1886	15.2%	9.72	Q1
2	Journal of Clinical Oncology	616	5.00%	33.0	Q1
3	Annals of Oncology	369	2.98%	18.3	Q1
4	Breast Cancer Research and Treatment	340	2.74%	3.83	Q2
5	PLOS One	276	2.23%	2.74	Q2
6	European Journal of Cancer	191	1.54%	7.28	Q1
7	Modern Pathology	176	1.42%	5.99	Q1
8	Scientific Reports	165	1.33%	3.99	Q3
9	Breast	163	1.31%	3.75	Q2
10	Breast Cancer Research	159	1.28%	4.99	Q1

### Research Hotspots: Co-Word Analysis and Clustering Analysis of Keywords

As a general overview of the literature theme, keywords are highly refined and generalized to a specific topic and can fully interpret the literature. Using high-frequency keywords to elucidate the research hotspots in a discipline can effectively determine the research hotspots and other important issues.

The literature search identified 12,429 TNBC-related publications and extracted 11,535 keywords with BICOMB. The frequency of the 50^th^ word was equal to its ordinal number, so terms ranked above 50 could be defined as high-frequency keywords (**Table 3**). The top 10 most frequent keywords after excluding the keywords without actual referential meanings are prognosis, apoptosis, metastasis, epithelial-mesenchymal transition, chemotherapy, biomarker, immunotherapy, neoadjuvant chemotherapy, *Brca1*, and survival. We constructed the binary matrix (**Supplementary Table 2**) and co-word matrix (**Supplementary Table 3**) based on high-frequency keywords. Subsequently, gCLUTO was used for biclustering analysis, and the mountain and heat maps were drawn based on this. Additionally, VOSviewer was used for visualization analysis of the keywords that co-occurred at least 15 times or more.

**Table 3:** High-frequency Keywords in the TNBC Study.

Rank	Keywords	Frequency, n	Percentage, %	Cumulative Percentage, %
**1**	Triple negative breast cancer	3044	10.0	10.0
**2**	Breast cancer	1842	6.08	16.1
**3**	Prognosis	421	1.39	17.5
**4**	Triple negative	351	1.16	18.7
**5**	Apoptosis	339	1.12	19.8
**6**	Metastasis	315	1.04	20.8
**7**	Epithelial-mesenchymal transition	195	0.64	21.5
**8**	Chemotherapy	189	0.62	22.1
**9**	Biomarker	150	0.49	22.6
**10**	Immunotherapy	149	0.49	23.1
**11**	Neoadjuvant chemotherapy	144	0.48	23.6
**12**	Brca1	127	0.42	24.0
**13**	Survival	124	0.41	24.4
**14**	Immunohistochemistry	121	0.40	24.8
**15**	Invasion	120	0.40	25.2
**16**	Targeted therapy	120	0.40	25.6
**17**	Egfr	117	0.39	26.0
**18**	Proliferation	101	0.33	26.3
**19**	Migration	95	0.31	26.6
**20**	Cancer	92	0.300	26.9
**21**	Estrogen receptor	90	0.30	27.2
**22**	HER2	89	0.29	27.5
**23**	Autophagy	89	0.29	27.8
**24**	Molecular subtype	86	0.28	28.1
**25**	Androgen receptor	80	0.26	28.3
**26**	Cancer stem cells	79	0.26	28.6
**27**	PD-L1	76	0.25	28.9
**28**	Angiogenesis	76	0.25	29.1
**29**	MDA-MB-231	70	0.23	29.3
**30**	Breast neoplasms	70	0.23	29.6
**31**	MicroRNA	69	0.23	29.8
**32**	Doxorubicin	69	0.23	30.0
**33**	Paclitaxel	69	0.23	30.2
**34**	PARP inhibitor	66	0.22	30.5
**35**	Metastatic breast cancer	65	0.21	30.7
**36**	Pathological complete response	65	0.21	30.9
**37**	Cisplatin	65	0.21	31.1
**38**	Cell cycle	63	0.21	31.3
**39**	Akt	62	0.20	31.5
**40**	Tumor-infiltrating lymphocyte	62	0.20	31.7
**41**	Overall survival	58	0.19	31.9
**42**	Brain metastases	58	0.19	32.1
**43**	P53	57	0.19	32.3
**44**	Stat3	56	0.18	32.5
**45**	Breast	55	0.18	32.7
**46**	Tumor microenvironment	53	0.17	32.8
**47**	Drug resistance	52	0.17	33.0
**48**	Basal-like breast cancer	51	0.17	33.2
**49**	Cancer stem cells	51	0.17	33.3
**50**	Triple negative breast neoplasms	50	0.17	33.5

**Figure 3:**
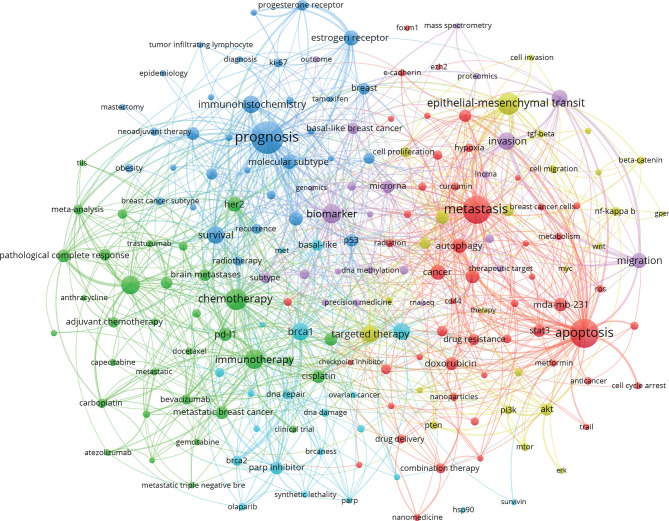
Keyword Co-occurrence Map Based on VOSviewer in TNBC research field.

Keyword co-occurrence analysis refers to counting the frequency of appearance of keywords in the same literature and analyzing the intrinsic relationships and degree of intimacy among keywords. Based on this, closely related keywords are grouped into different clusters through clustering analysis. These clusters reflect the key research contents and core research fields that the keywords refer to (21). To ensure a visual effect and analysis emphasis, any keywords without actual referential meanings were excluded from this article, and the keywords that appeared at least 15 times were selected for visualization. A total of 186 keywords appeared at least 15 times, and the co-occurrence map of keywords was drawn (**Figure 3**). The nodes in the map indicated the corresponding keywords, and the size of node indicated how many publications in TNBC field included the corresponding keywords. The bigger the node size, the greater the popularity of the keyword. The link line between 2 keyword nodes indicated the relationships between the keywords. The keywords, such as prognosis, metastasis, apoptosis, had many link lines with other nodes, indicating that these keywords have a close relationship with other keywords in this field (**Figure 3**).

**Figure 4:**
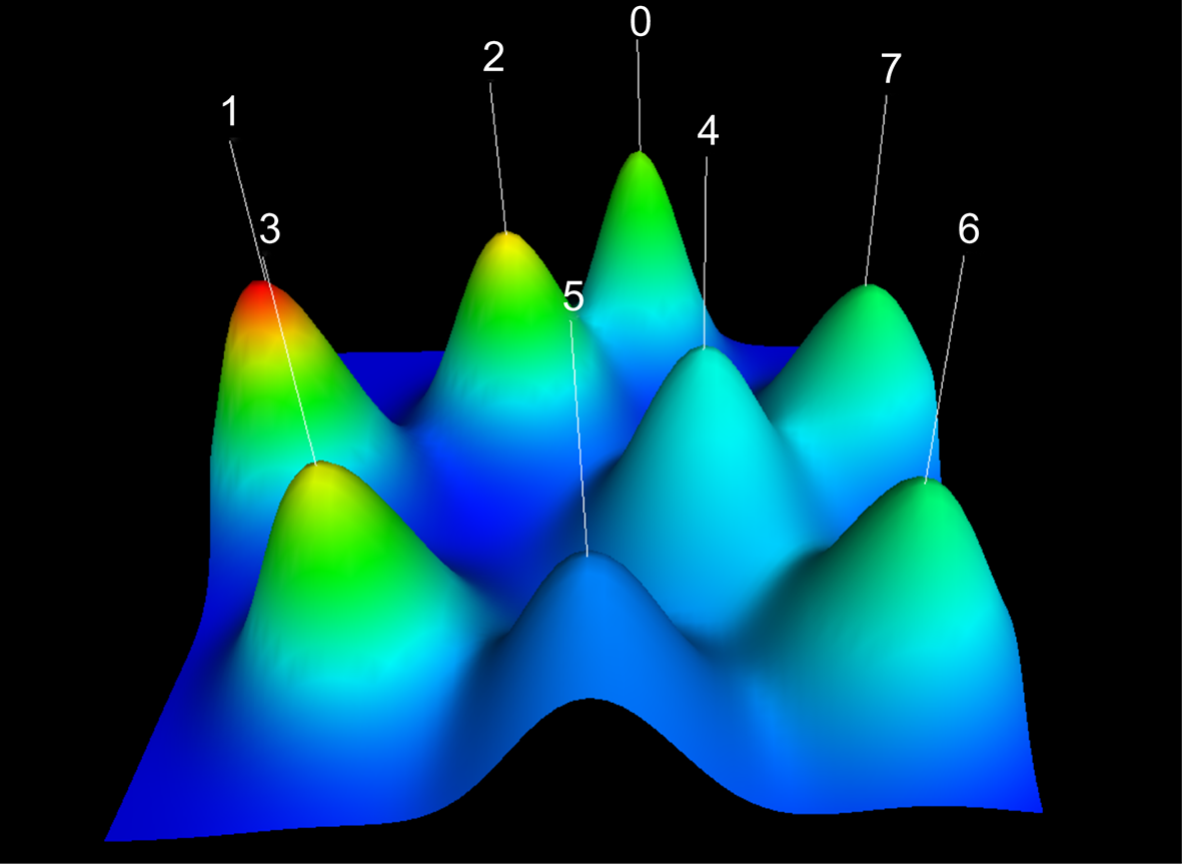
Visualized Mountain Map based on the Biclustering analysis of TNBC Binary Matrix of Word-paper. Cluster 0: Immunotherapy for TNBC. Cluster 1: The role of EMT in TNBC tumor cell metastasis; Cluster 2: The effect of neoadjuvant chemotherapy in TNBC treatments; Cluster 3: The Application of Paclitaxel in TNBC Treatments; Cluster 4: PARP inhibitors in TNBC treatments. Cluster 5: Tumor stem cell studies of TNBC; Cluster 6: Tumor microenvironment of TNBC; Cluster 7: Molecular subtypes of TNBC.

In the visualized mountain map (**Figure 4**), the marked number corresponds to the cluster number. The volume of the mountain is directly proportional to the number of keywords within the cluster, and the height is also directly proportional to the intra-class similarity of the cluster. A sharp peak signifies high intra-class similarity. The peaks are shown in five colors: red, yellow, green, light blue, and dark blue. The standard deviation of the intra-class similarities represented by these colors increased in turn. The distance between the peaks was used to evaluate the similarity between the two clusters. The eight peaks were relatively independent and clearly distributed, indicating a satisfactory clustering effect.

**Figure 5:**
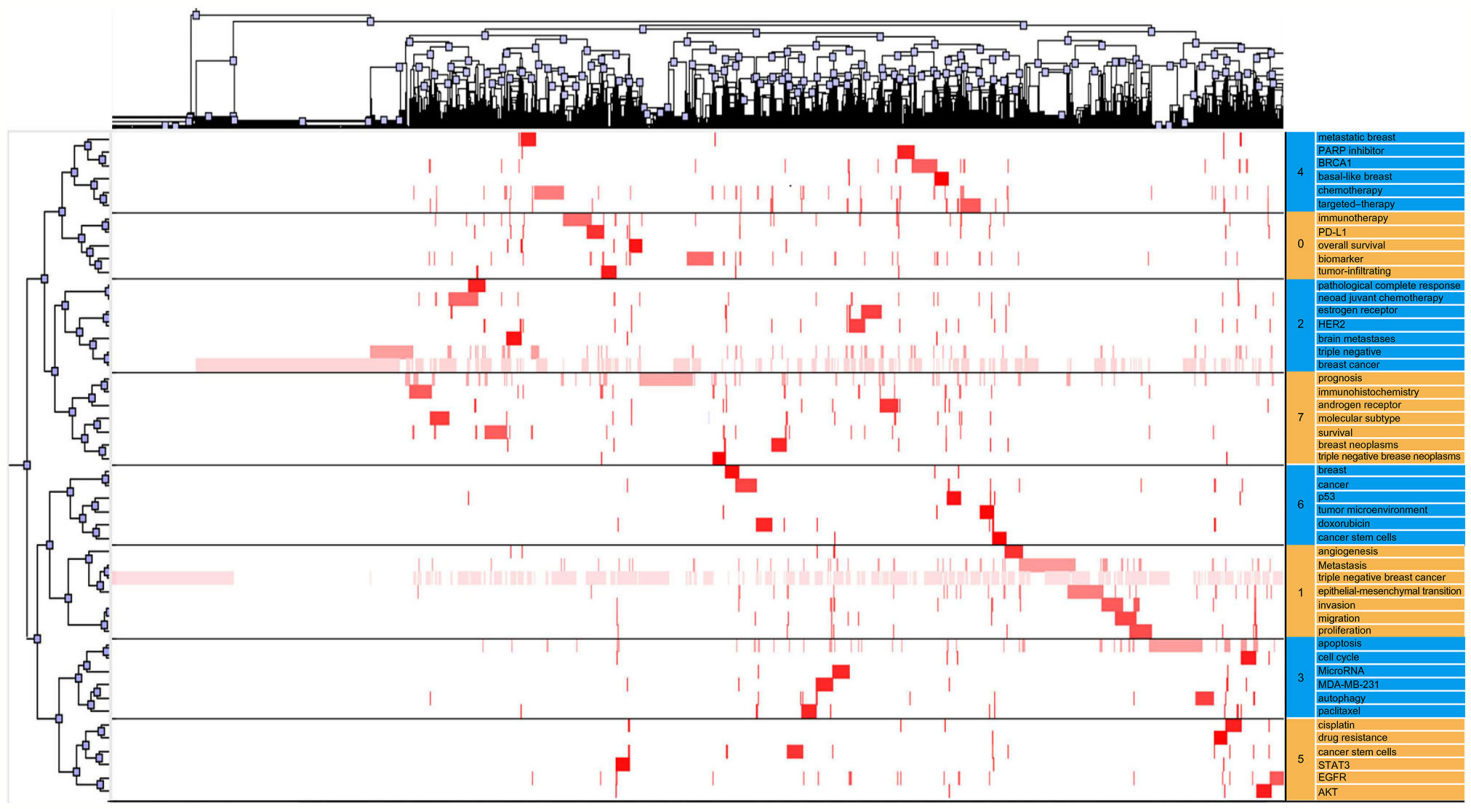
Visualized matrix based on the biclustering analysis of TNBC binary matrix of Word-paper.

In the visualized heat map (**Figure 5**), the rows represent high-frequency words and columns represent published literature. The colors represent values in the original data matrix. The values in the original data matrix are represented by color depth. The white area in the figure represents a value close to zero. The gradually deepened red area represents larger values. The clustering tree represents article clusters containing high-frequency words. By identifying the semantic connections among high-frequency words and their source articles, we confirmed eight research hotspots in the TNBC research field:

Cluster 0: Immunotherapy for TNBC.
Cluster 1: The role of EMT in TNBC tumor cell metastasis.
Cluster 2: The effect of neoadjuvant chemotherapy in TNBC treatments.
Cluster 3: The Application of Paclitaxel in TNBC Treatments.
Cluster 4: PARP inhibitors in TNBC treatments.
Cluster 5: Tumor stem cell studies of TNBC.
Cluster 6: Tumor microenvironment of TNBC.
Cluster 7: Molecular subtypes of TNBC.

### Strategic Diagram Analysis

Based on the gCLUTO blustering analysis, we calculated each cluster’s centrality and density (**Supplementary Table 4**) (22). The coordinates of each cluster were obtained accordingly. With “centrality” as the abscissa and “density” as the ordinate, we considered the mean value of centrality and density from all clusters as the origin of the coordinate (7.62, 3.05) and drew the strategic diagram. Each point in **Supplementary Figure 2** represents a cluster. The coordinate value of each cluster is the difference between centrality/density and their mean values. As shown in **Supplementary Figure 2**, the above eight clusters were distributed in four different quadrants. We then analyzed the research status of various hot topics on TNBC. The clusters in the first quadrant (Cluster 2, neoadjuvant chemotherapy, Cluster 3, Paclitaxel and Cluster 7 molecular subtypes) had high centrality and density and close internal and extensive connections with the rest of the clusters, indicating the maturity of these three types of research and their core positions in the research field. Though in a marginal position, a fairly completed research system has taken shape in the cluster of the second quadrant (Cluster 1, EMT). The centrality and density of clusters in the third quadrant (Cluster 5, tumor stem cell, and Cluster 6, special tumor microenvironment) were low. The internal connections of the research topic are not closely related to each other, and connections with other groups are relatively loose, which puts them on the edge of the whole research field. The clusters in the fourth quadrant (Cluster 0, immunotherapy and Cluster 4, PARP inhibitor) were lower in density but higher in centrality, indicating that the studies of immunotherapy and PARP inhibitors for TNBC are not yet mature but are the current focus of this field. These emerging topics will be the future trend of TNBC research. We usually focus on hot topics in the fourth quadrant. It should be noted that the locations of various clusters in strategic diagrams are not fixed. The maturity of certain research themes or the emergence of new knowledge points may lead to a transfer of the first quadrant to the second quadrant. Similarly, with the development of themes in the second and third quadrants, they also have the potential to move to the first quadrant.

### Research Fronts: Keyword Burst Analysis

**Figure 6:**
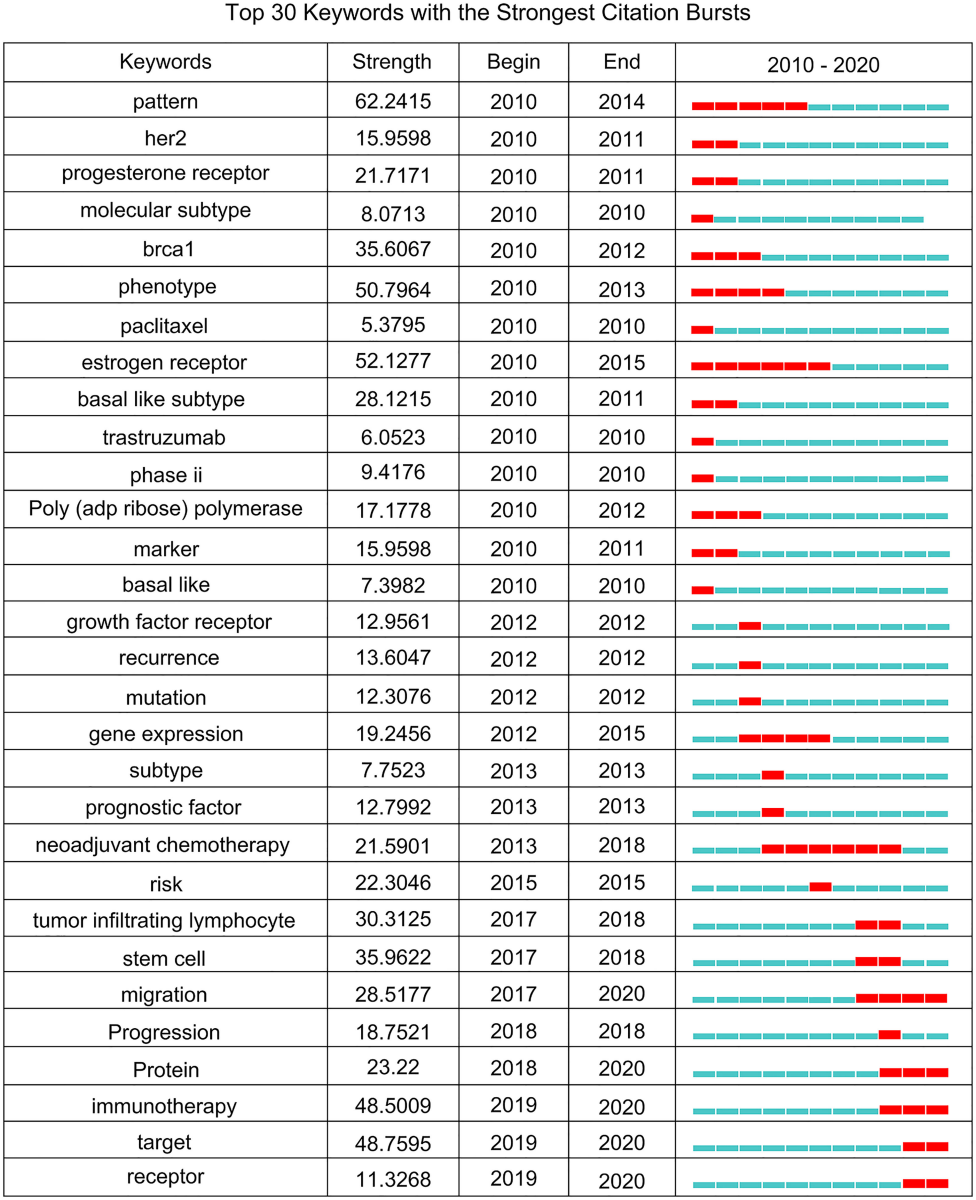
The evolution trend of burst words on TNBC from 2010 to 2020.

Research fronts are the newest and most potential research topics or fields in scientific research. The burst of keywords reflects the sudden increase in a citation in a certain period, displays the time distribution and dynamic variability of keywords, and accurately reveals the evolution trend of hotspots in the research field (17, 25). Additionally, burst detection of keywords is also helpful in finding keywords that have not reached the frequency threshold but may have academic contributions to analyze the hotspots and fronts of TNBC research more comprehensively. The stronger the burst intensity, the greater the attention the research topic arises and the more it reflects research published during a given period. We detected keywords of 12,429 TNBC articles from 2010 to 2020 using the burst detection algorithm of CiteSpace. In the diagram generated by CiteSpace, the timeline was shown as a blue line, and the interval during which a keyword showed a burst was depicted as a red segment in a specific location of the blue timeline. The specific burst words, burst strengths, and starting and ending years are indicated. The top 30 keywords with the highest burst strength are shown in **Figure 6**.

The TNBC research field presents a diversified characteristic, and different burst words appear in different periods. The keywords with the strongest intensity were “Pattern” (62.2415), followed by “Estrogen Receptor” (52.1277) and “Phenotype” (50.7964). The bursts of these 3 keywords all began in 2010 and last for 3-5 years. In the recent three years, the keywords with the strongest intensity were “Target” (48.7595) and “Immunotherapy” (48.5009). The keywords with the longest burst time were “Estrogen Receptor” (2010 - 2015) and “Neoadjuvant Chemotherapy” (2013 - 2018). It is noteworthy that the five keywords bursting in the past two years were receptor (2019 - 2020), target (2019 - 2020), immunotherapy (2019 - 2020), protein (2018 - 2020), and migration (2017 - 2020). This indicates that these research themes are relatively active in recent TNBC studies and may become research fronts in the future.

## Discussion

In the present study, we analyzed the publications of triple negative breast cancer between 2010 and 2020 using information visualization methods. A total of 12,429 articles related to TNBC were identified. During 2010-2020, the most productive country/region in TNBC field was the USA, followed by China and Italy. The University of Texas MD Anderson Cancer Center had the maximum number of publications in TNBC field, followed by Dana-Farber Cancer Institute, Fudan University, and Memorial Sloan Kettering Cancer Center. Cancer Research, Journal of Clinical Oncology, and Annals of Oncology were the first three periodicals with maximum publications in TNBC research. Co-word analysis and clustering analysis of keywords identified eight research hotspots in TNBC field, that is, Cluster 0: Immunotherapy for TNBC; Cluster 1: The role of EMT in TNBC tumor cell metastasis; Cluster 2: The effect of neoadjuvant chemotherapy in TNBC treatments; Cluster 3: The application of Paclitaxel in TNBC treatments; Cluster 4: PARP inhibitors in TNBC treatments. Cluster 5: Tumor stem cell studies of TNBC; Cluster 6: Tumor microenvironment of TNBC; and Cluster 7: Molecular subtypes of TNBC.

### Cluster 0: The Immunotherapy for TNBC

Breast cancer has always been considered a “cold tumor” with low immunogenicity. However, more studies have found that due to a high genomic instability and mutational burden of TNBC (26), the expression levels of the programmed cell death ligand 1 (PD-L1) protein are high, and the tumor-infiltrating lymphocytes (TIL) are rich in the microenvironment. Thus, it is assumed that breast cancer might be a “hot tumor” with a positive immune response (27).

PD-L1 inhibitors are currently the most thoroughly studied and widely used immune checkpoint inhibitors (28). Although PD-L1 expression is also observed in primary breast cancer, it is more prevalent in TNBC (20% to 30%). The immune escape mechanism of TNBC makes it more suitable for immune checkpoint blockade therapy (29, 30). Although TNBC had these characteristics that enhance anticancer immune responses, the single-agent efficacy of immune checkpoint inhibitors in TNBC is low (31). Combination regimens of PD-1/L1 inhibitors plus chemotherapy have demonstrated more success in metastatic TNBC than single-agent PD-1/L1 inhibitors. In March 2019, following the results of IMpassion130, a phase III clinical trial, the combined application of the PD-L1 antibody atezolizumab and the albumin-binding paclitaxel was approved by the FDA as the first-line therapy for metastatic or unresectable locally advanced TNBC (32, 33). This was the first immunotherapy approved for breast cancer. This therapy achieved clinically significant overall survival benefits in PD-L1-positive TNBC patients, and most adverse events (AEs) were lowered (34). This was followed by the KEYNOTE-355 study which showed that pembrolizumab in combination with chemotherapy had a significant and clinically meaningful improvement in PFS versus placebo-chemotherapy among patients with metastatic TNBC with CPS of 10 or more (35). Based on results of KEYNOTE-355, pembrolizumab + chemotherapy was approved by FDA in November 2020 to treat patients with locally recurrent unresectable or metastatic TNBC expressing PDL1. It is noteworthy that introducing immune checkpoint inhibitors in the early stages of TNBC may be a potential therapy because the primary tumor seems more immunogenic than the metastatic tumor (36). These results indicate that TNBC treatment has entered the era of immunotherapy.

Additionally, many studies have shown significant infiltration of TIL in TNBC, and high levels of TIL are significantly associated with a reduced distant recurrence rate of primary TNBC (37). Moreover, TILs in TNBC are strong independent indicators of prognosis, and the extended disease-free survival and overall survival periods can be touted (38, 39).

Immunotherapy has brought a new hope and option for TNBC patients, which is expected to alter the existing clinical treatment standard for advanced TNBC. In coming years, more biomarkers should be explored to accurately screen the population benefiting from single-agent immunotherapy and improve the prognosis (29, 33, 38, 40). Besides, novel therapeutic strategies to overcome a lack in anticancer immunity in TNBC are urgently needed and likely to be a research focus in future.

### Cluster 1: The Role of EMT in TNBC Tumor Cell Metastasis

Unlike primary tumors, metastatic diseases are not curable because of their systemic nature and inoperable features. Therefore, the spread of breast cancer tumor cells and eventual distant metastasis (mainly lung, bone, and brain) are clinical problems to be resolved. Despite standardized adjuvant chemotherapy, the 5-year survival rate of patients with metastatic TNBC was still less than 30% (41, 42). Metastasis occurs through a series of complex cellular biological events, among which the spread of tumor cells to distant organs is one of the most critical steps.

Currently, it is well accepted that the epithelial-mesenchymal transition (EMT) is a critical mechanism for the initiation of tumor cell metastasis in TNBC. EMT is a process wherein cells lose their epithelial features and gain mesenchymal features. The loss of connections and apical-basal polarity of epithelial cells, as well as cytoskeletal reorganization, occurs in this process, which increases the activity and aggressiveness of the cells (43). However, EMT is believed to limit cell migration and promote colonization and growth of metastasized tumor cells. Recent results suggest that EMT is not a complete transition from epithelial to mesenchymal state but a transition state between the two, and this state is reversible (44), that is, EMT and its reverse process, mesenchymal-epithelial transition (MET), are both dynamic. The mesenchymal cancer cells are likely to undergo MET transiently and subsequently re-undergo EMT to restart the metastatic process. Cells in this state have a high metastatic potential. They can effectively invade blood vessels to enter the systemic circulation and easily colonize the distant organs (45), which means such bidirectional transitions between epithelial and mesenchymal cells are involved in cancer development. EMT is a potential therapeutic target for TNBC. Specific anti-EMT drugs may be effective in preventing tumor metastasis in the future.

### Cluster 2: The Effect of Neoadjuvant Chemotherapy on TNBC

Currently, neoadjuvant chemotherapy (NACT) is a conventional treatment for early-stage TNBC. Compared with adjuvant chemotherapy, NACT reduces the tumor burden before surgery. It also allows for further assessment of the prognosis of the tumor and its response to chemotherapy for subsequent adjuvant chemotherapy plans accordingly. Despite the substantial high rate of recurrence, TNBC patients have a more pronounced response to NACT compared with other subtypes of breast cancer patients, which is known as the “TNBC paradox” (46). Thus, the risk of tumor recurrence is high without chemotherapy, but the benefit is greater after treatment.

Currently, the standard regimen of NACT is still a combination of anthracyclines and taxanes. Approximately 30–40% of early-stage TNBC patients prescribed this therapy can achieve pathological complete response (PCR) (47, 48). Additionally, platinum-based compounds (as DNA damage agents) show better efficacy when tumor cells have DNA repair defects (such as the *BRCA 1/2* gene mutations). Incidentally, the mutation frequency in germline *BRCA* in TNBC patients is higher than that of other subtypes. Thus, platinum drugs exhibit promising clinical results in TNBC patients (7). Many trials have explored the effects of platinum in neoadjuvant settings, and the current consensus is that the application of platinum-based on standard chemotherapy will enhance the PCR of TNBC patients at the cost of noticeably increased chances of level 3/4 hematological AEs (49, 50). Therefore, exploration of new adjuvant chemotherapy regimens with platinum as the main component, which has less toxicity and side effects, has broad prospects and should be further investigated.

### Cluster 3: The Application of Paclitaxel in TNBC Treatments

Paclitaxel (PTX) is an antimitotic chemotherapy drug widely used in a variety of cancers. By stabilizing microtubules, the cell cycle stops at the G2 and M stages, leading to subsequent apoptosis (51). Paclitaxel is widely used in many cancers and is currently the first-line chemotherapy drug for TNBC. Although traditional paclitaxel has good efficacy, its further clinical use is limited due to its poor solubility and toxic side effects (mainly peripheral neuropathy).

However, albumin-bound paclitaxel (nab-PTX) solves this problem considerably. Nab-paclitaxel is a modified structure based on traditional paclitaxel. By utilizing albumin nanoparticles as carriers, the safety and efficacy of paclitaxel are improved (52). Furthermore, cancer cells often overexpress albumin binding glycoprotein SPARC (an acid-secreting protein rich in cysteine), which promotes the release of drugs in tumor regions for a better targeted antitumor effect (53). Moreover, compared with solvent-based paclitaxel, nab-paclitaxel does not need a co-solvent; thus, the related hypersensitivity reactions are avoided. Problems such as preventive application of glucocorticoids and excessively long infusion time are also solved. It is predicted that nab-paclitaxel will become a research hotspot in the future.

### Cluster 4: PARP Inhibitors in TNBC Treatments

The lack of suitable therapeutic alternatives for TNBC in the past is largely due to the lack of therapeutic targets. *BRCA* is currently the most vital tumor suppressor gene related to the occurrence and development of breast cancer, which plays a crucial role in repairing damaged DNA and maintaining genomic stability. The incidence of the *BRCA1/2* pathogenic mutation in TNBC patients is 10–20% (54, 55). In addition to *BRCA1/2*, DNA single-strand breaks (i.e., DNA damage) are repaired by PARP. When PARP inhibitors are used upon an existing *BRCA1/2* mutation, DNA repair in tumor cells is further limited, thereby resulting in lethality, i.e., the “synthetic lethality” phenomenon, which is the treatment principle of PARP inhibitors (56).

The OlympiAD clinical trial phase III showed that when compared with the chemotherapy group, the progression-free survival (PFS) of HER2-negative breast cancer patients (with *gBRCA* mutations) was prolonged by 2.8 months following administration of PARP inhibitors, olaparib. The clinical efficacy and safety of olaparib have been confirmed (57, 58). Based on this study, the FDA approved olaparib as treatment for these patients.

It is necessary to employ *BRCA* gene detection screenings among the appropriate population for early identification of those patients sensitive to PARP inhibitors. Precision therapy based on molecular characteristics of TNBC patients is the future direction of therapeutic development.

### Cluster 5: A Tumor Stem Cell Study of TNBC

Reya et al. first proposed the term “tumor stem cells” in 2001, suggesting that malignant tumor tissues have a small number of cell subsets that retain stem cell features. These cells are called tumor stem cells (59). They have the ability of self-renewal, infinite proliferation, and multidirectional differentiation, which are related to tumor occurrence and recurrence.

Currently, most solid tumors, including breast cancer, are believed to be stem cell diseases (60). Compared with other subtypes, a high proportion of tumor stem cells in TNBC is considered an important factor of adverse outcomes. It is usually assumed that the recurrence of malignant tumors after a series of conventional treatments is due to the surviving tumor stem cells. During chemotherapy, surviving tumor stem cells are selectively enriched in the residual tumor, which differentiate into fast proliferating cells insensitive to drugs. Thus, they supplement the tumor cells lost during chemotherapy, resulting in chemotherapy resistance and tumor progression (61). Moreover, breast cancer stem cells (BCSCs) have strong migration abilities. Compared with other solid tumors, BCSCs are more likely to break away from the primary site, migrate, and invade lymphatics or blood vessels, causing breast cancer metastasis (62). Additionally, CSCs are highly tumorigenic. Previous trials have shown that 100 breast cancer tumor cells with stem cell phenotypes transplanted into non-obese diabetic/severe combined immunodeficiency mice can regenerate tumors, and the new tumor has all the histopathological characteristics of the original tumor (63).

Accumulative studies have shown that TNBC cells show CSCs signatures at functional, molecular, and transcriptional levels. For example, the CD44+/CD24- phenotype and high ALDH activity have become the “golden standard” signature for BCSCs after research of Al-Hajj et al. and Ginestier et al. (61, 64). Interestingly, histopathological analyses revealed that TNBC tissues had more enriched CD44+/CD24- and ALDH1 expression signatures compared to non-TNBC tissues (65, 66), suggesting that the TNBC phenotype is highly like the CSC phenotype. In addition, the EMT signature, which can ultimately facilitate tumor cell migration, is consistently observed in both TNBC and CSCs cells (67). These data collectively indicated that BCSCs are enriched in TNBC, which may contribute to the propensity of TNBC for tumor metastasis and chemotherapy resistance, providing a different insight into the aggressive nature of TNBC. In the future, CSCs in research will focus on the role of CSCs in the tumor biology of TNBC to develop new, effective targeted therapies and improve prognosis of TNBC patients.

### Cluster 6: The Special Tumor Microenvironment of TNBC

In 1889, Stephen Paget first proposed the “seed and soil” theory, wherein cancer cells were “seeds” and the microenvironment was the “soil” for their occurrence and metastasis. The tumor microenvironment (TME) is composed of vascular endothelial cells, mesenchymal stem cells (MSCs), tumor-associated fibroblasts (CAFs), immune cells, and extracellular matrix, which induce tumor proliferation, inhibit cell apoptosis, stimulate angiogenesis, and tumor immunosuppression, thereby blocking the antitumor response of TNBC and promoting its occurrence and development (68).

The excessive proliferation of tumor cells and abnormal vascular structure may lead to a hypoxic microenvironment. Consequently, endothelial cells are stimulated to generate new branch vessels that provide oxygen and nutrients, as well as a pathway for tumor metastasis. CAFs account for the highest proportion of stromal cells in TME. When activated by tumor cells, CAFs secrete various growth factors and chemokines. The former promotes growth and metastasis of tumor cells, while the latter guides recruitment of various types of extracellular matrix cells (69). In the TME, immune surveillance and immune escape mechanisms of tumor cells and the human immune system work against each other. Immune cells from various families show antitumor and tumorigenesis manifestations upon receiving environmental signals in the TME (70). It has been found that cytotoxic CD^8+^ T lymphocytes and CD4+ T lymphocytes in the TME induce antitumor immunity and are independent and favorable prognostic factors (71). However, most tumor-associated macrophages (TAMs) in the TME have the M2 phenotype, supporting tumor angiogenesis and metastasis.

Current research on TME is limited. TNBC has a unique immune microenvironment, and research on therapeutic targets of TME will contribute to early diagnosis and effective treatment of TNBC. Unfortunately, there is no standard treatment strategy for TME-specific components in TNBC patients.

### Cluster 7: The Molecular Subtypes of TNBC

TNBC is especially characterized by extensive genomic, cellular, and phenotypic heterogeneity. There is no unified standard for molecular typing of TNBC, and the Lehmann classification system is the earliest and most mature TNBC typing system at present. In 2011, Lehmann’s team conducted a detailed analysis of breast cancer gene expression profiles, revealing that the so-called “triple negative” cancer was just a common manifestation of a complex heterogeneity of multiple types of TNBC. Thus, TNBC could be specifically divided into six subtypes as follows: basal-like 1 (BL1), basal-like 2 (BL2), immunomodulatory (IM), mesenchymal (M), mesenchymal stem-like (MSL), and luminal androgen receptor (LAR) (23). BL-1 TNBC is primarily characterized by the lack of cell cycle regulation and impaired DNA damage repair machinery, and this subtype is highly sensitive to platinum chemotherapy drugs and presents the best prognosis. In the BL-2 subtype, the growth factor signaling pathway is abnormally active, and both the basal subtypes show high expression levels of proliferation-related genes. The M and MSL subtypes are related to cell movements and show high expression levels of EMT and stem cell-associated genes. The LAR TNBC subtype is associated with a high mutation burden and poor prognosis. This type of cell line depends on androgen growth and is sensitive to androgen receptor inhibitors such as bicalutamide and enzalutamide. Patients with the IM subtype exhibit high levels of immune signaling and checkpoint gene expressions, and they are most likely to benefit from treatment with checkpoint inhibitors.

In 2016, Lehmann et al. discovered that the gene expression profile characteristics of the IM and SLM subtypes were related to tumor-infiltrating lymphocytes and surrounding stromal cells, respectively. Thus, TNBC subtypes were grouped into four categories: BL-1, BL-2, M, and LAR. It is confirmed that different subtypes have significant heterogeneity in several aspects, including the age of onset, degree of malignancy, treatment sensitivity, and prognosis (72).

Based on different detection methods and purposes, other common subtypes include Burstein subtypes and Fudan subtypes (73, 74). In addition, a plethora of high-dimensional technologies, such as single cell RNA sequencing and spatial transcriptomics, has provided new insights into the understanding of subclonal diversity of TNBC (75). Single cell RNA-seq allows the assessment of gene expression patterns at an individual cell level and may provide stronger power to identify tumor cell subpopulations that drive poor prognosis. For instance, using a new single-cell, single-molecule DNA-sequencing method called acoustic cell tagmentation, Minussi et al. observed that there was a period of transient genomic instability followed by ongoing copy number evolution during expansion of primary tumor mass after early evolutionary events including clonal *TP53* mutations, genome doubling and extensive loss-of-heterozygosity events. Furthermore, by expanding single daughter cells *in vitro*, they found that TNBCs quickly rediversify their genomes into multiple subclones and do not retain isogenic properties. These results suggested that during primary expansion of TNBC, the chromosomal aberrations occur continuously and TNBC cells maintain a reservoir of subclonal diversity (76). Karaayvaz1 et al. used single cell RNA-seq and found a single subpopulation which was associated with several signatures of metastasis and treatment resistance. This subpopulation was characterized functionally by activation of glycosphingolipid metabolism and associated innate immunity pathways (77). Moreover, the Lindeman group described three epithelial subsets including luminal progenitor, basal stem/progenitor, and mature luminal cells from precancerous breast tissues of individuals heterozygous for a *BRCA1* mutation and normal mammary tissues. The *BRCA1^mut/+^* tissue harbored an aberrant luminal progenitor population which showed a markedly higher *in vitro* clonogenic activity compared with normal breast tissues. Besides, breast tissues heterozygous for a *BRCA1* mutation and basal breast tumors were more similar to normal luminal progenitor cells in gene expression profile than any other subset, including the stem cell-enriched population, indicating that the basal-like subclass of breast tumors might be progressed from luminal progenitor (78). In the future, studies may focus on uncovering additional cell subpopulations and elucidating how they govern tumor behavior, particularly with respect to non-malignant compartments.

Keyword bursts may indicate the frontier topics or emerging trends in a certain field. In the selected years, the research on pattern, *Brca1*, phenotype and estrogen receptor showed a strong burst at the beginning (2010), and then several keywords, such as growth factor receptor and reoccurrence, showed a citation burst during 2012-2017, yet the bursts were not strong. In the recent 3 years, the keywords immunotherapy and target showed a prominent burst, which were the keywords we were particularly interested in. We have discussed the immunotherapy for TNBC in cluster 0. “Target” is a very generic word which usually refers to therapeutic target in the context of TNBC research. From the summary of keywords with high frequency in TNBC study (**Table 3**) and keyword burst detection results (**Figure 6**), therapeutic target has always been a research focus since keywords such as EGFR, PARP inhibitor, PD-L1 all had high frequency, and they showed a burst at different beginning year during 2010-2020. These suggested that identification of therapeutic targets for TNBC management is throughout the TNC studies, and therefore, it seems necessary to continue to investigate this issue.

However, there are some limitations in our study. First, we only retrieved publications from the WoS Core Collection. Therefore, not all relevant publications were included in this study. Second, although the database is constantly updated, we only included publications from January 2010 to October 2020, which may cause exclusion of some latest research results.

## Conclusion

In this article, we summarized knowledge on TNBC from a visualization and bibliometric perspective. We focused on eight hotspots in TNBC research, which were summarized using bibliometric analysis. At the core of the hotspots, the NACT and paclitaxel therapy for TNBC treatment, as well as the molecular subtypes of TNBC are relatively mature. However, immunotherapy of TNBC, PARP inhibitors, and other targeted therapies are not yet mature, making them a future trend of this research field. Furthermore, “migration”, “protein”, and “receptors” are still very popular TNBC burst words among researchers and will continue to be the research direction in the future. Further studies on these topics may help improve our understanding of the pathogenesis of TNBC and guide its treatment.

## Data Availability Statement

The original contributions presented in the study are included in the article/**Supplementary Material**. Further inquiries can be directed to the corresponding author.

## Author Contributions

K-jH and XJ conceived of the study and designed the study. W-tD, K-jH, and XJ analyzed data and wrote the initial draft of the manuscript. All authors contributed to the article and approved the submitted version.

## Conflict of Interest

The authors declare that the research was conducted in the absence of any commercial or financial relationships that could be construed as a potential conflict of interest.

## Publisher’s Note

All claims expressed in this article are solely those of the authors and do not necessarily represent those of their affiliated organizations, or those of the publisher, the editors and the reviewers. Any product that may be evaluated in this article, or claim that may be made by its manufacturer, is not guaranteed or endorsed by the publisher.

## Supplementary Material

Supplementary Figure 1 The core author and intellectual basis of TNBC Research Field. **(A)** The visualization network of co-cited authors; **(B)** The visualization network of co-cited articles.

Supplementary Figure 2 The strategic diagram based on results of biclustering analysis and co-word matrix.
