# Supplementary figures and images for: Mapping Intellectual Structures and Research Hotspots of Triple Negative Breast Cancer: A Bibliometric Analysis
Source: Front Oncol. 2022 Jan 3;11:689553. doi: 10.3389/fonc.2021.689553 (PMC8763010; doi:10.3389/fonc.2021.689553)

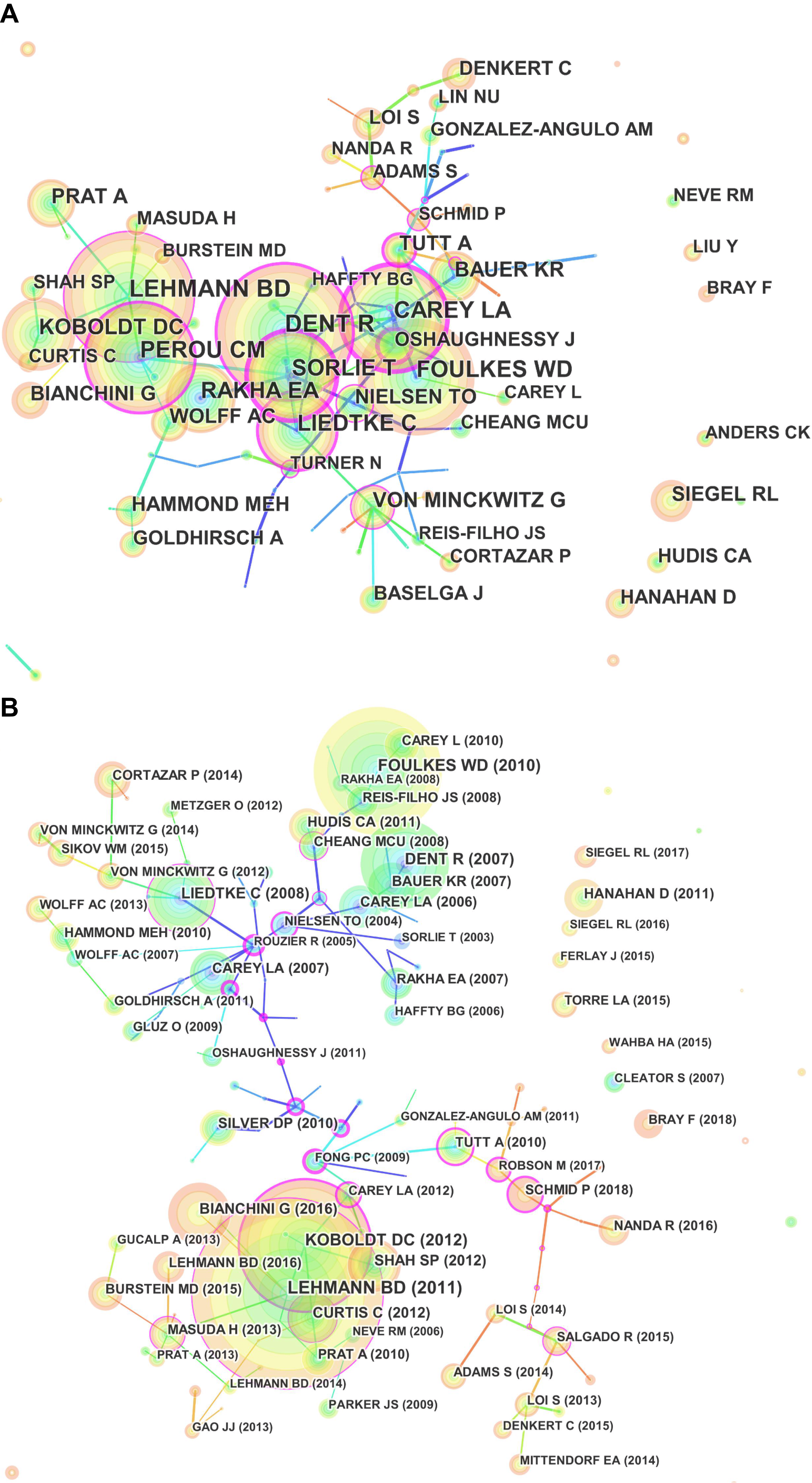

Supplement: Supplementary Figure 1 — The core author and intellectual basis of TNBC Research Field. (A) The visualization network of co-cited authors; (B) The visualization network of co-cited articles. [file Image_1.tif]

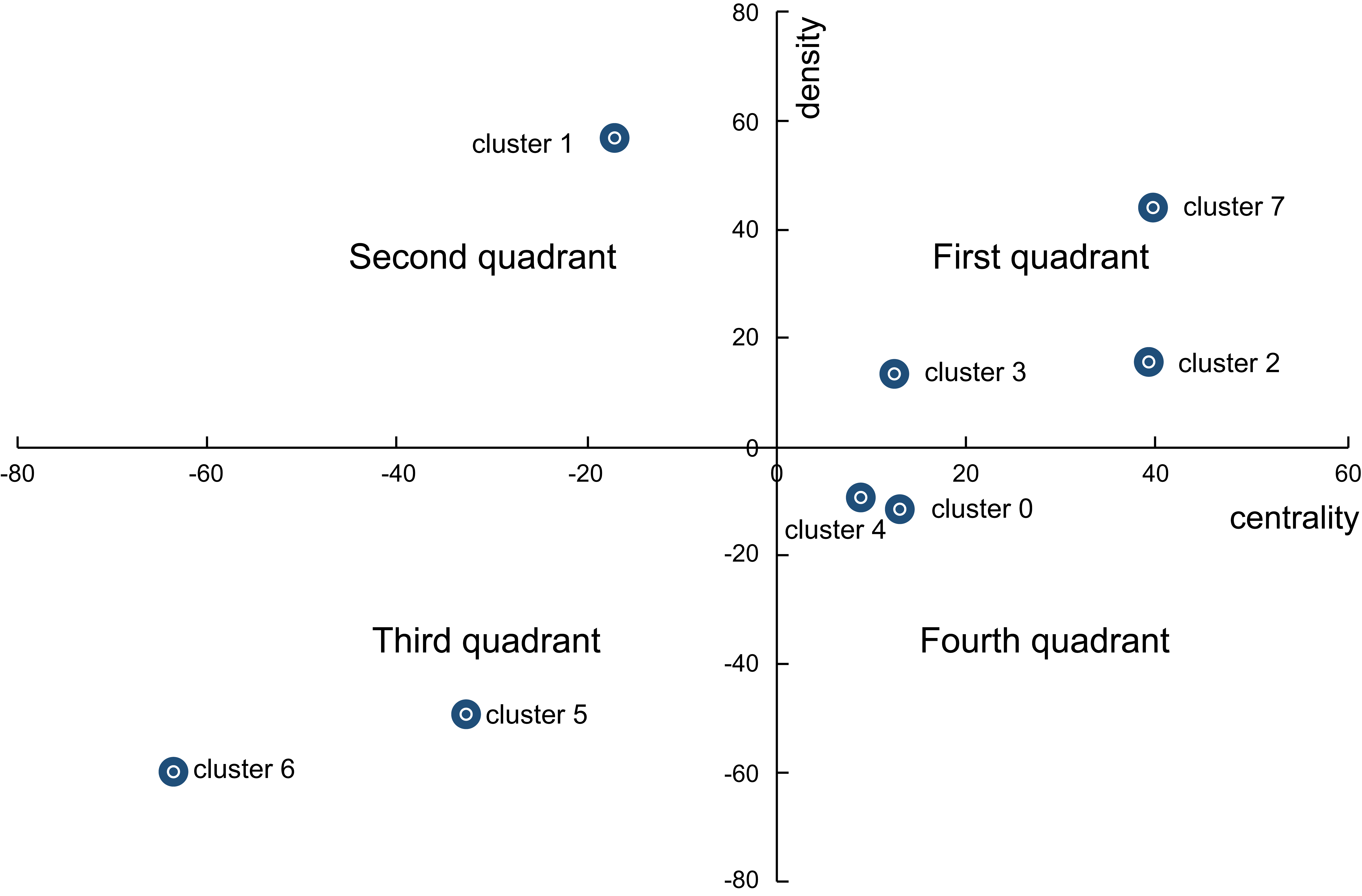

Supplement: Supplementary Figure 2 — The strategic diagram based on results of biclustering analysis and co-word matrix. [file Image_2.tif]
