# Supplementary material for: Mapping Intellectual Structures and Research Hotspots of Triple Negative Breast Cancer: A Bibliometric Analysis
Source: Front Oncol. 2022 Jan 3;11:689553. doi: 10.3389/fonc.2021.689553 (PMC8763010; doi:10.3389/fonc.2021.689553)
Supplement: Supplementary file 3 [file Table_1.docx]

**Supplementary Table 1 The top 10 most procreative authors who contributed to publications and the top 10 co-cited authors and references in TNBC research**

| **Rank** | **Author** | **Frequency** | **Co-cited Authors** | **Frequency**  **n** | **Top 10 References** | **Frequency n** |
| --- | --- | --- | --- | --- | --- | --- |
| 1 | Zhang J | 105 | DENT R | 1495 | Lehmann BD, 2011, J CLIN INVEST, V121, P2750 | 1189 |
| 2 | Liu Y | 99 | LEHMANN BD | 1462 | Koboldt DC, 2012, NATURE, V490, P61 | 822 |
| 3 | Wang Y | 98 | FOULKES WD | 1345 | Foulkes WD, 2010, NEW ENGL J MED, V363, P1938 | 807 |
| 4 | Shao ZM | 93 | PEROU CM | 1257 | Dent R, 2007, CLIN CANCER RES, V13, P4429 | 555 |
| 5 | Wang J | 93 | CAREY LA | 1197 | Liedtke C, 2008, J CLIN ONCOL, V26, P1275 | 427 |
| 6 | Zhang Y | 92 | SORLIE T | 1019 | Bianchini G, 2016, NAT REV CLIN ONCOL, V13, P674 | 395 |
| 7 | Li J | 89 | LIEDTKE C | 873 | Shah SP, 2012, NATURE, V486, P395 | 347 |
| 8 | Ueno NT | 85 | RAKHA EA | 860 | Curtis C, 2012, NATURE, V486, P346 | 322 |
| 9 | Pusztai L | 80 | KOBOLDT DC | 831 | Bauer KR, 2007, CANCER, V109, P1721 | 304 |
| 10 | Winer EP | 80 | BAUER KR | 636 | CAREY LA, 2007, CLIN CANCER RES, V13, P2329 | 289 |

**Supplementary Table 2 Binary matrix table of TNBC high-frequency keywords and articles**

| No | Keyword |  |  | Paper ID |  |  |  |
| --- | --- | --- | --- | --- | --- | --- | --- |
|  |  | **001** | **002** | **003** | **…** | **12424** | **12425** |
| 1 | TNBC | 0 | 0 | 0 | … | 1 | 0 |
| 2 | Breast cancer | 0 | 0 | 0 | … | 1 | 1 |
| 3 | Prognosis | 0 | 0 | 0 | … | 0 | 0 |
| 4 | Triple negative | 0 | 0 | 0 | … | 0 | 0 |
| 5 | Apoptosis | 0 | 1 | 0 | … | 0 | 0 |
| … | … | … | … | … | … | … | … |
| 49 | Cancer stem cells | 0 | 0 | 0 | …- | 0 | 0 |
| 50 | Triple negative breast neoplasms | 0 | 0 | 0 | … | 0 | 0 |

**Supplementary Table 3 Co-word matrix table of TNBC high-frequency keywords**

| No. | Keyword | TNBC | Breast cancer | Prognosis | … | Triple negative breast neoplasms |
| --- | --- | --- | --- | --- | --- | --- |
| 1 | TNBC | 3044 | 325 | 220 | … | 0 |
| 2 | Breast cancer | 325 | 1842 | 148 | … | 1 |
| 3 | Prognosis | 220 | 148 | 421 | … | 11 |
| … | … | … | … | … | … | … |
| 50 | Triple negative breast neoplasms | 0 | 1 | 11 | …- | 50 |

**Supplementary Table 4 The strategic coordinate parameters of the eight clusters**

| Cluster | Intra-class link averages | Density-Y | Inter-class link averages | Centrality-X |
| --- | --- | --- | --- | --- |
| 0 | 120.1999999 | -11.5005952 | 148.8 | 13.0154762 |
| 1 | 188.5714286 | 56.87083337 | 118.7142857 | -17.07023809 |
| 2 | 147.2857143 | 15.58511909 | 175.0 | 39.2154762 |
| 3 | 145.1666667 | 13.46607147 | 148.1666667 | 12.38214287 |
| 4 | 122.3333333 | -9.367261867 | 144.6666667 | 8.882142867 |
| 5 | 82.5 | -49.2005952 | 103.1666667 | -32.61785713 |
| 6 | 71.8333333 | -59.86726187 | 72.3333333 | -63.45119047 |
| 7 | 175.7142857 | 44.01369051 | 175.4285714 | 39.64404763 |
